# Beneficial effects of benznidazole in Chagas disease: NIH SaMi-Trop cohort study

**DOI:** 10.1371/journal.pntd.0006814

**Published:** 2018-11-01

**Authors:** Clareci Silva Cardoso, Antonio Luiz P. Ribeiro, Claudia Di Lorenzo Oliveira, Lea Campos Oliveira, Ariela Mota Ferreira, Ana Luiza Bierrenbach, José Luiz Padilha Silva, Enrico Antonio Colosimo, João Eduardo Ferreira, Tzong-Hae Lee, Michael P. Busch, Arthur Lawrence Reingold, Ester Cerdeira Sabino

**Affiliations:** 1 School of Medicine, Federal University of São João del-Rei, Divinópolis, Brazil; 2 Hospital das Clínicas, Universidade Federal de Minas Gerais, Belo Horizonte, Brazil; 3 Institute of Tropical Medicine, University of São Paulo, São Paulo, Brazil; 4 Hospital das Clínicas, State University of Montes Claros, Montes Claros, Brazil; 5 Hospital Sirio-Libanês, São Paulo, Brazil; 6 Department of Statistics, Universidade Federal do Paraná, Curitiba, Brazil; 7 Blood Systems Research Institute and University of California, San Francisco, California, United States of America; 8 Department of Epidemiology, University of California, Berkeley, California, United States of America; University of Texas at El Paso, UNITED STATES

## Abstract

**Background:**

The effectiveness of anti-parasite treatment with benznidazole in the chronic Chagas disease (ChD) remains uncertain. We evaluated, using data from the NIH-sponsored SaMi-Trop prospective cohort study, if previous treatment with benznidazole is associated with lower mortality, less advanced cardiac disease and lower parasitemia in patients with chronic ChD.

**Methods:**

The study enrolled 1,959 ChD patients and abnormal electrocardiogram (ECG) from in 21 remote towns in Brazil. A total of 1,813 patients were evaluated at baseline and after two years of follow-up. Those who received at least one course of benznidazole were classified as treated group (TrG = 493) and those who were never treated as control group (CG = 1,320). The primary outcome was death after two-year follow-up; the secondary outcomes were presence at the baseline of major ChD-associated ECG abnormalities, NT-ProBNP levels suggestive of heart failure, and PCR positivity.

**Results:**

Mortality after two years was 6.3%; it was lower in the TrG (2.8%) than the CG (7.6%); adjusted OR: 0.37 (95%CI: 0.21;0.63). The ECG abnormalities typical for ChD and high age-adjusted NT-ProBNP levels suggestive of heart failure were lower in the TrG than the CG, OR: 0.35 [CI: 0.23;0.53]. The TrG had significantly lower rates of PCR positivity, OR: 0.35 [CI: 0.27;0.45].

**Conclusion:**

Patients previously treated with benznidazole had significantly reduced parasitemia, a lower prevalence of markers of severe cardiomyopathy, and lower mortality after two years of follow-up. If used in the early phases, benznidazole treatment may improve clinical and parasitological outcomes in patients with chronic ChD.

**Trial registration:**

ClinicalTrials.gov, Trial registration: NCT02646943.

## Author summary

Chagas disease remains as one of the most neglected infectious diseases of the world, with millions of persons infected and dozens of thousands of deaths every year. The disease has a very long natural history and few options of etiologic treatment, including antiparasitic [1] drugs benznidazole and nifurtimox. Benznidazole has been recommended for treatment of the disease based mostly in physiopathological assumptions and relatively small observational studies and, recently, a negative trial was published, showing the absence of benefit of benznidazole in patients with Chagas cardiopathy. Thus, there is an urgent need not only of new therapeutic options but also of further evidence of the utility of benznidazole, at least in those patients with less severe disease. We conducted an observational study in the NIH SaMi-Trop cohort comparing patients with self-reported previous treatment with benznidazole with non-treated ones, using as primary outcome death in the follow-up of 2 years, and as secondary outcomes markers of severity based in the ECG and NT-proBNP levels, as well as parasitemia by PCR for Trypanosoma cruzi DNA in blood. Patients previously treated with benznidazole had significantly reduced parasitemia, a lower frequency of markers of severe cardiomyopathy and reduced mortality. If used in the early phases of Chagas disease, benznidazole treatment may result in better clinical and parasitological outcomes. It is reasonable to consider treating all Chagas disease patients without advanced cardiopathy with benznidazole, especially those less than 50 years-old of age.

## Introduction

Chagas disease (ChD), caused by the protozoa *Trypanosoma cruzi*, is one of the most important neglected diseases and a leading cause of cardiopathy and death in Latin America, [1][2] where it is endemic with an estimated 5.7 million infected people [3]. With continuing migration from Latin America to North America and Europe, ChD has become a global problem, with hundreds of thousands of infected persons living in non-endemic countries [1]. Global costs related to ChD are estimated at $7.19 billion per year, with a substantial proportion of the burden resulting from lost productivity from cardiovascular disease-induced morbidity and early mortality [4].

ChD has a very long natural history which begins that an acute phase, generally a benign and self-limited febrile disease in childhood [2]. After remission of the acute manifestations, most patients enter a chronic phase without clinical manifestations of the disease, the so-called chronic indeterminate form of ChD, which can persist for decades or be lifelong, or can evolve into the cardiac or intestinal forms of the disease [1]. Chagas cardiomyopathy is the most serious manifestation of ChD and may present as heart failure, ventricular arrhythmias, heart block, or thromboembolic phenomena [1]; ~2% of indeterminate form patients evolve to clinically diagnosed cardiac disease each year [5]. The pathophysiological mechanisms underlying and prognostic markers predicting progression to cardiomyopathy are poorly understood.

Two drugs with proven anti-parasite efficacy that are used to treat ChD patients are available: nifurtimox and benznidazole; most studies have focused on the use of benznidazole due to its better tolerability, reduced toxicity and, possibly, enhanced efficacy [6]. In Brazil, only BZN has been approved for commercialization. It treatment schedule recommended is 300mg/day for 60 consecutive days [7].

Although persistence of low-level systemic infection, including cardiac *T*. *cruzi* infection in the indeterminate phase seems to play an important role in the development of Chagas cardiomyopathy, the effectiveness of treatment during the chronic phase in preventing progression of disease and in reducing mortality remains unclear [8] [9]. Indeed, although observational studies have suggested that treating chronic ChD patients with benznidazole can lead to parasite clearance reversion, and reduced progression of clinical manifestation [8] [10], the recently published BENEFIT trial, a multicenter, randomized, double-blinded, placebo-controlled trial, did not demonstrate a beneficial effect of benznidazole on cardiac outcomes in patients with chronic Chagas cardiopathy [9]. The role of benznidazole in preventing the appearance of cardiac lesions when used in less advanced disease is still controversial. In this observational study, conducted in a large sample of Brazilian ChD patients, we evaluated if previous treatment with benznidazole is associated with less advanced cardiac disease, lower prevalence of detectable parasitemia and lower mortality, compared to untreated control patients with chronic ChD.

## Methods

### Study design, population measurements and outcomes

SaMi-Trop is a prospective cohort study with two-year follow-up to date (recently funded to continue follow-up), including one baseline visit and another visit at 24 months [11]. The cohort was established with patients under the care of the Telehealth Network of Minas Gerais (TNMG), a program designed to support primary care in Brazil [12]. In this publicly funded program, all patients’ electrocardiograms (ECG) and clinical data are sent to a central reading unit that also collects clinical data, such as the history of the diagnosis of ChD. Using the database from 2011 and 2012, we selected 21 municipalities within a limited region in the northern part of the State of Minas Gerais, where the prevalence of ChD is high. Patients who reported having ChD at the time of their previous ECG examination in 2011 and 2012 were invited to join this cohort.

**Fig 1:**
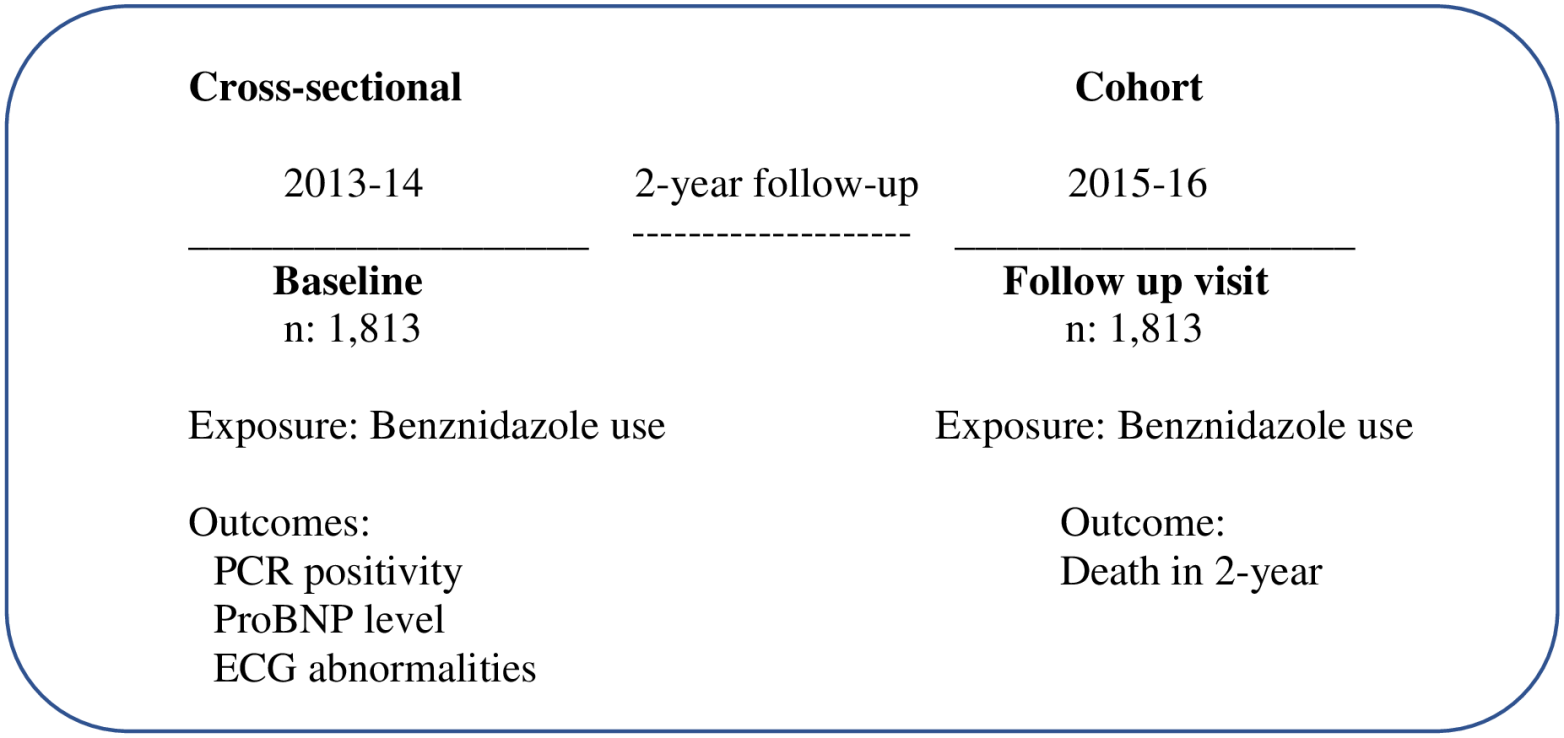
Study design diagram.

For this investigation, we used two epidemiological study designs. The first was a cross-sectional analysis using baseline SaMi-Trop data collected at recruitment time (2013–14) and the second one was a prospective cohort analysis using only death as an outcome collected at the end of two-year of follow-up (2015–16, see Fig 1).

The exposition was self-reported treatment with benznidazole, which occurred (or not) several years before the recruitment for the study. The primary outcome was death from any cause after two-year follow-up (2015–16). Secondary outcomes were all evaluated at baseline of SaMi-Trop study (2013–14) and included the presence of typical ChD-associated ECG abnormalities, age-adjusted brain-type natriuretic peptide (NT-ProBNP) levels suggestive of heart failure, and polymerase-chain-reaction (PCR) positivity for *T*. *cruzi* DNA in blood. We also developed a composite outcome in which we combined ECG abnormalities typical for ChD and age-adjusted NT-proBNP levels suggestive of heart failure.

At baseline, all consenting participants were interviewed, with detailed questions on the duration and clinical manifestations of ChD and previous use of benznidazole, and blood was collected for *T*. *cruzi* serology, PCR and NT-proBNP measurement. To confirm the self-disclosed ChD diagnosis, serum samples from all enrolled patients were tested for the presence of anti-*T*. *cruzi* antibodies using a chemiluminescent microparticle immunoassay (Architect Chagas, Abbott Laboratories, Wiesbaden Germany). Negative results were confirmed by two other enzyme immunoassays (EIA) presenting different *T*. *cruzi* antigens (Chagatest v.4, Wiener and Chagas, Diasorin). The final study population for the current analyses consisted only of patients confirmed to be *T*. *cruzi* seropositive [11]. The target-capture (TC) real-time (RT) PCR assay used in this study was developed based on the method described by Pyron et al. [13]. Two replicate TC-PCR assays were performed and results interpreted as positive if both were positive. If only one replicate result was positive, it was considered as inconclusive, and another aliquot of this sample was processed in four replicates. The results were considered positive if at least two of the four additional replicates were positive [14]. NT-proBNP (Roche diagnostics) was categorized according to age-specific cut-points for heart failure [15] [16].

An ECG was also performed at the baseline visit. Although a report of an abnormal ECG was considered for recruitment of patients using the telehealth database, 17% of these ECG did not fulfill diagnostic criteria of the presence of an ECG abnormality after standardized central reading. The resting 12-lead ECG was recorded using a PC-based ECG machine (TEB, São Paulo, Brazil). The ECG recordings were sent electronically to the Telehealth system and the ECG measurements were obtained through automated analysis using the University of Glasgow ECG analysis program (release 28.5, issued on January 2014) [17]. All ECGs were over-read manually by an experienced cardiologist. It were classified using the Minnesota Code (MC) criteria, with variables derived from the median complex of the Glasgow University software measurement matrix [18]. ECG abnormalities considered typical of Chagas cardiomyopathy included [17]: complete intraventricular block (7.1, 7.2, 7.4 or 7.8), frequent ventricular premature beats (MC 8.1.2 or 8.1.3), atrial fibrillation or flutter or supraventricular tachycardia (MC 8.3.x. or 8.4.2), other major arrhythmias (MC 8.2.x, except 8.2.1), major atrioventricular conduction abnormalities or pacemaker use (MC 6.1, 6.2.x, 6.4, 6.8, 8.6.1 or 8.6.2), major Q-wave abnormalities [MC 1.1.x or 1.2.x] or minor Q-waves abnormalities with ST segment or T-wave abnormalities [1.3.x and 4.1.x, 4.2, 5.1 or 5.2], or major isolated ST segment or T-wave abnormalities (MC 4.1.x, 4.2, 5.1 or 5.2).

Death during the 2-year follow-up period was ascertained for enrolled participants who could not be contacted for their 2-year follow-up visit by interviewing family members and reviewing death certificate data of the Mortality Information System database for the State of Minas Gerais.

The simple size was calculated considering the minimal number of events per variable acceptable in a proportional hazards regression analysis of 10 events per variable. For a 2-year follow-up period and annual mortality rate of 5% in chronic Chagas cardiomyopathy (10% in 2 years), the calculated sample size was about 2000 participants [11].

### Statistical analyses

ChD subjects who received at least one course of treatment with benznidazole were classified as belonging to the treated group (TrG = 493) and those who were never treated as control group (CG = 1,320). Patients who did not know whether they had been treated with benznidazole use were excluded from analysis (n = 146). In descriptive analyses, categorical variables were presented as numbers and percentages, continuous variables were summarized as means and standard deviations.

Three measures of treatment effectiveness were calculated. The first was an unadjusted effect measure (difference of proportions, averages or odds ratio, depending on the outcome being studied) using an unpaired t-test or Fisher’s exact test. The second comparison estimated the treatment effect using linear or logistic regression, adjusted for sex, age, income, literacy, duration of known ChD, and hypertension as covariates. The third approach was a pairing method (Genetic Matching), an evolutionary algorithm that seeks to maximize the balance of the distributions of the treated units and controls [19] [20], using the same covariates described above. This method can be used as an approximation of a traditional clinical trial and provides the basis to an inference of causality.

In the paired analysis (Genetic Matching), for the continuous outcomes, the standard test of the Matching package was used by means of *Abadie-Imbe*ns estimator for the standard error. Missing values in the covariates were treated by simple imputation; with this approach, there was a loss of six observations. The level of significance was α = 0.05, and the hypothesis tests were 2-sided. Analyzes were performed in R version 3.3.2 using GenMatch and Geepack packages.

We performed Kaplan-Meier survival analysis and used the log-rank test to compare time to death between treatment and control groups. Because the number of patients after two years of follow-up was small, we censored the follow-up after this time.

### Ethics statement

The study was conducted using data from the NIH-sponsored São Paulo-Minas Gerais Tropical Medicine Research Center (SaMi-Trop) study, established in a highly endemic region of Brazil [11]. The protocol was approved by the Brazilian National Institutional Review Board (CONEP), number 179.685/2012. In this investigation, all human subjects were adult whose have given written informed consent. Participants had full right to continue or withdraw from the study. The confidentiality of all participants was maintained throughout the study.

## Results

### Study patients

This investigation was conducted on the 1,813 patients who were confirmed as being infected with *T*. *cruzi* based on positive serology and who had information about benznidazole use, from a total of 2157 patient who were enrolled in the SaMi-Trop cohort. Previous use of benznidazole (TrG) was reported by 493 patients; the average duration of use was self-reported as 90 days. Most patients started treatment soon after their serological diagnosis of infection (64% within 30 days, 20% between 1–12 months, and 16% after one year). A total of 83% of the treated patients reported that were treated before having 40 years of age. The characteristics of the patients according to treatment group are presented in Table 1.

**Table 1:** Characteristics of the patients, by treatment and control group.

Characteristics	Valid number	Treatment (n: 493)	Control (n: 1320)	P-value
Age—mean (SD)	1813	54.1±11.5	59.4±12.5	<0.001
Female sex—n (%)	1813	337/493 (68.4)	893/1320 (67.7)	0.755
Illiterate—n (%)	1807	149/493 (30.2)	600/1314 (45.7)	<0.001
Family monthly income—US$	1306	370.87±203.04	417.39+209.56	<0.001
Duration of ChD > 10 yr—n (%)	1768	355/484 (73.3)	728/1284 (56.7)	<0.001
Hypertension—n (%)	1813	280/493 (56.8)	852/1320 (64.5)	<0.001
Diabetes mellitus—n (%)	1813	34/493 (6.9)	133/1320 (10.1)	<0.001
Pacemaker—n (%)	1792	24/493 (5.0)	86/1299 (6.6)	0.291
Medication—n (%)				
Diuretic	1790	195/487 (40.0)	670/1303 (51.4)	<0.001
Digoxin	1795	22/487 (4.5)	104/1308 (8.0)	<0.001
Amiodarone	1749	87/464 (18.8)	324/1283 (25.3)	<0.001
Beta-blocker	1791	41/486 (8.4)	91/1305 (7.0)	0.210
ACE inhibitor	1790	128/487 (26.3)	381/1303 (29.2)	0.150
ARA	1791	125/487 (25.7)	373/1304 (28.6)	0.153
ECG typical ChD—n (%)	1779	233/491 (47.3)	773/1288 (60.0)	<0.001
High age-adjusted NT-ProBNP n (%)	1810	30/492 (6.1)	175/1318 (13.3)	<0.001
PCR positivity—n (%)	1813	82/493 (16.6)	481/1320 (36.4)	<0.001
Mortality in 2-year follow-up	1813	14/493 (2.8)	100/1320 (7.6)	<0.001

ACE = Angiotensin converting enzyme; ARA = angiotensin receptor antagonists; ECG = Electrocardiogram; NT-ProBNP = N-terminal of the prohormone brain natriuretic peptide; PCR = Polymerase-chain-reaction.

The mean age was higher for the CG (CG: 59.1 years versus TrG: 54.1 years, P<0.01). Most of the patients were female and the majority in both groups self-reported having been diagnosed with ChD for more than 10 years (TrG: 73.3% versus CG: 58.4%) before being interviewed. In general, patients in the CG presented with worse clinical conditions, including hypertension, diabetes mellitus, ECG typical of Chagas disease, NT-ProBNP levels suggestive of heart failure and PCR positivity, as well as higher use of medications other than benznidazole (P<0.01), except Beta-blockers, ACE and ARA.

### Primary and secondary outcomes

**Fig 2:**
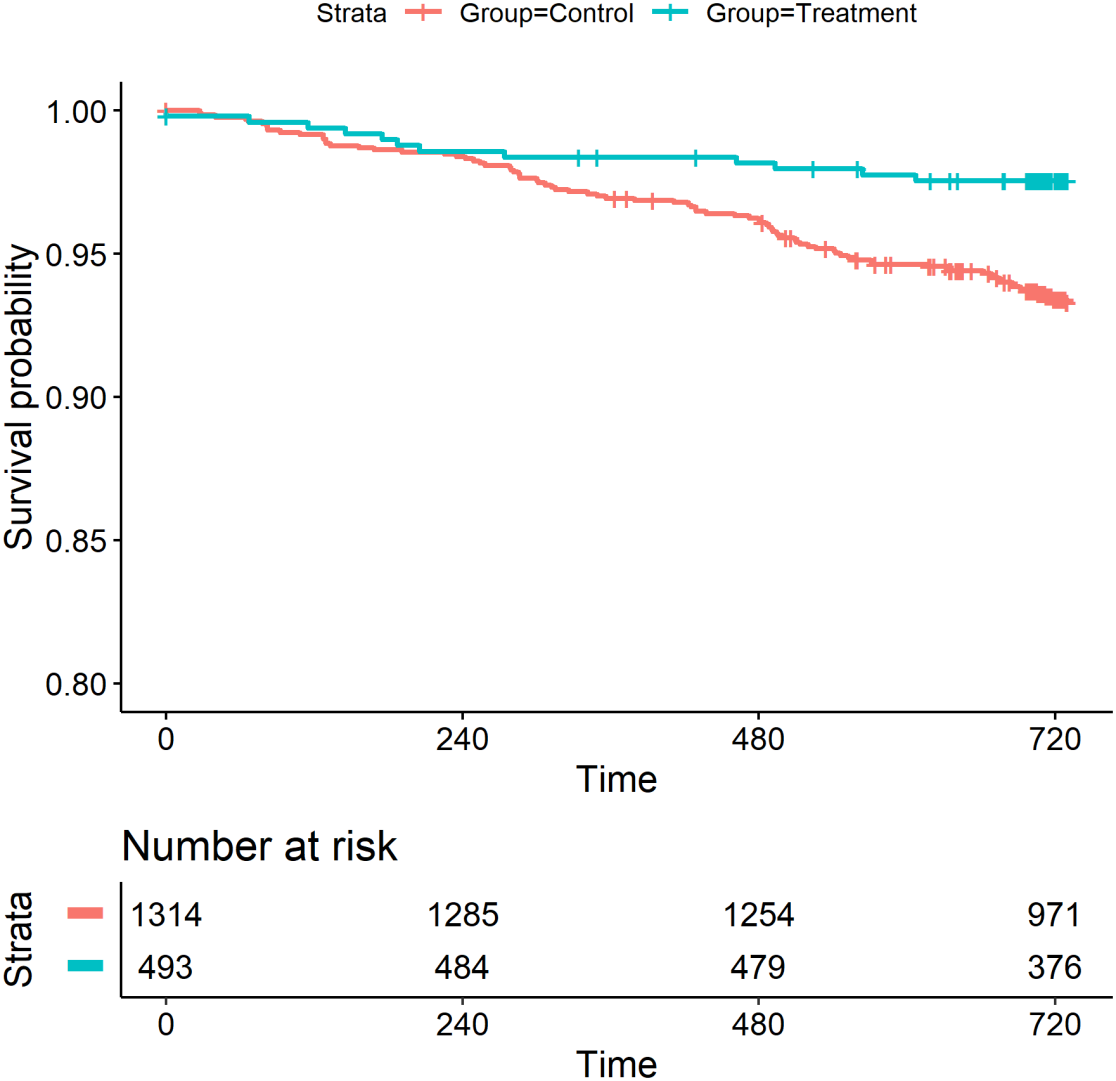
Kaplan-Meier survival curves in ChD by treatment and control group.

Adverse outcomes in the TrG unmatched-control (unadjusted or adjusted by linear or logistic regression), and matched-control (genetic matching) analysis are presented in Table 2. All reported adjusted odds ratios (OR) in this section used the genetic matching method, but similar effect of treatment was observed using adjusted regression. Proportions or means of key clinical and parasitological results at the index visit were lower in the TrG than in the CG (P<0.01). The overall mortality after two years of follow-up in enrolled patients with positive serology for ChD was 114/1,813 (6.3%). It was lower in the TrG (2.8%, 14/493) than in the CG (7.6%, 100/1,320); adjusted OR: 0.37 (0.21,0.63), indicating protection against death in the TrG. Survival differed significantly among treatment and control groups: patients treated with benznidazole had better survival compared with those in the CG (P = 0.001, Fig 2).

**Table 2:** General outcome in the treatment, unmatched and matched control groups.

Outcomes	Treatment % (n)	Control % (n)	Univariate	Multivariate (Regression)*	Genetic Matching (matched analysis)
			OR^†^ (95% CI)	OR (95% CI)	OR (95% CI)
Mortality 2-year follow-up	2.8 (14/493)	7.6 (100/1320)	0.36 (0.19, 0.63)	0.42 (0.23, 0.76)	0.37 (0.21, 0.63)
High age-adjusted NT-ProBNP	6.1 (30/492)	13.3 (175/1318)	0.42 (0.27, 0.64)	0.41 (0.27, 0.63)	0.41 (0.28, 0.60)
ECG abnormalities typical ChD	48.4 (233/481)	60.0 (773/1288)	0.63 (0.50, 0.78)	0.67 (0.53, 0.84)	0.64 (0.52, 0.79)
ECG abnormalities typical ChD + High age-adjusted NT-ProBNP	5.0 (24/480)	12.6 (162/1286)	0.37 (0.22, 0.57)	0.36 (0.23, 0.57)	0.35 (0.23, 0.53)
PCR positivity for *T*. *cruzi* DNA	16.6 (82/493)	36.4 (481/1320)	0.35 (0.26, 0.45)	0.35 (0.26, 0.46)	0.35 (0.27, 0.45)

NT-ProBNP = N-terminal of the prohormone brain natriuretic peptide; ECG = Electrocardiogram; ChD = Chagas disease; PCR = Polymerase-chain-reaction.

* Adjusted by age, gender, literate, duration of ChD, family monthly income and hypertension.

^†^Odds ratio

The age-adjusted NT-proBNP levels were lower in the TrG (6.1%, 30/492) than in CG (13.3%, 175/1,318), indicating an adjusted odds ratio was 0.41 (95%CI: 0.28; 0.60) in favor of the TrG. The TrG had a lower frequency of patients with NT-proBNP levels, above age-adjusted reference values (Table 2). Major ECG abnormalities typical of ChD were lower in the TrG (48.4% versus 60.0%), yielding an adjusted OR of 0.64 (CI: 0.52; 0.79). The TrG had a smaller proportion of worse combined adverse outcomes (ECG abnormalities typical of ChD and age-adjusted NT-proBNP levels) than the CG: 5.0% (24/480) *versus* 12.6% (162/1286); adjusted OR: 0.35 (CI: 0.23, 0.53). *T*. *cruzi* PCR was positive in blood samples from 16.6% of TrG patients *versus* 36.4% of the CG patients, with an adjusted OR using GenMatch of 0.35 [CI: 0.27; 0.45].

## Discussion

In this investigation, when comparing patients previously treated with benznidazole with similar controls, we observed a lower frequency of markers of ChD cardiomyopathy severity a reduction in parasitemia and a significantly lower risk of mortality over a 2 years of follow-up period. Our findings help clarify the controversy about the effectiveness of benznidazole in the treatment of ChD, especially in the early chronic phase of the disease. Although there is an urgent need for better therapies than can stop or delay the progression of ChD in millions of infected persons around the world, [21] none of the new compounds tested for treating ChD thus far has come close to the efficacy observed with benznidazole [22].

A significant reduction in parasitemia, as measured by PCR, in patients treated with benznidazole has been reported in previous studies [10], including data from the REDS-2 study [23] and the BENEFIT trial [9]. Benznidazole has also been shown to be superior to posaconazole and E1224, a new anti-parasite drug, in reduction of PCR positivity in ChD patients, as recently shown in multicenter randomized placebo-controlled studies with parasitological endpoints, with follow-up periods of 10 to 12 months [24] [25] [26]. Our data from a large population in which most patients had been treated more than 10 years ago extends previous observations, as we show that PCR positivity for *T*. *cruzi* was twice in untreated patients as compared with treated ones, suggesting that benzonidazole induces a long-lasting clearance of parasitemia.

The most important contribution of the present work is our demonstration of a marked clinical benefit from benznidazole, including a reduction in well-established markers of ChD severity, such as typical ECG abnormalities, high NT-proBNP levels or both, as well as mortality during the two years of follow-up after recruitment. The presence of typical or major ECG abnormalities is an important marker of risk in Chagas disease [27] and marks the transition from the indeterminate to the cardiac form of the disease [1]. Viotti et al [28] and Fragata-Filho et al. [8] reported that treatment with benznidazole reduced the appearance of new ECG abnormalities in a long-term follow-up study. However, a meta-analysis published recently showed that this prevention of progression to cardiopathy was not significant when three smaller observational studies were jointly analyzed [10]. Furthermore, in the BENEFIT study, in which 94% patients had an abnormal ECG at baseline, the rate of development of new ECG abnormalities was the same in the treated and untreated groups [9].

Our results are further reinforced by the effect of previous treatment on NT-proBNP levels, as those who were treated with benznidazole had a significantly lower frequency of high age-adjusted levels than those who were untreated, both when NT-proBNP levels were considered as a single outcome or in association with presence of typical ECG findings. Indeed, high levels of BNP or NT-proBNP are an accurate marker of the presence of left ventricular systolic dysfunction [29] 6 and a strong prognostic marker in ChD patients [30]. The concomitance of typical ECG abnormalities and high NT-proBNP levels identifies a subgroup of patients with Chagas cardiopathy and left ventricular dysfunction, which is the group at highest risk of death. Thus, it is highly relevant that previous treatment with benznidazole is associated with a lower frequency of this composite outcome, which is itself a marker of an increased risk of death.

The SaMi-Trop study is the first to show that previous treatment with benznidazole is associated with a reduced risk of death in ChD. Viotti et. al observed a non-significant reduction in the rate of death in the treated group [28], but the study was too small to allow any conclusion about the beneficial effect of benznidazole in the risk of death. In the BENEFIT trial [9], there was no difference in the mortality rate between treated and untreated groups. Since 83% of the treated patients reported that the treatment occurred ≥ 10 years before the baseline evaluation, most patients in the cohort were treated before having 40 years of age, probably in an early stage of the development of the disease. In contrast, those recruited for the BENEFIT trial were, on average 55 years, of age and had well-established cardiomyopathy at the time treatment. These differences suggest that effectiveness of the benznidazole in preventing progression of cardiomyopathy and death related to ChD is dependent on the stage of the disease and the age of the patient when the treatment is initiated, which may help overcome reluctance about the use of benzonidazole in patients with earlier stages of the disease who are less than 50 years of age.

The present study had limitations. We do not have reliable information about the dose of trypanocidal drug or about the exact duration of treatment, as most patients used the drug more than 10 years before the study interview. In this manuscript as the duration of treatment was self-reported, we believe in memory bias, considering the long time between exposure and interview to collect precision information. However, because benznidazole usage is a very specific event in the life of a ChD patient, we believe that most patients accurately recalled the prior use of this medication as Yes/No. Moreover, we cannot assure that patients who received benznidazole had the same clinical profiles as those who did not receive treatment. If those treated had an average, milder disease than those who were untreated, our results could have been biased in favor of treatment effect. This limitation is inherent to the study design, although it is likely that, more than 10 years ago, when the treatment decisions were made, most patients would have had milder forms of the disease. Additionally, those with severe cardiopathy (at the time of the decision of treatment or not) probably did not survived the recruitment period, considering the ominous prognosis of severe Chagas cardiomyopathy. The imbalance in various features among the groups, typical of all observational studies, was at least partly corrected by the method of matching used in this study, as confirmed by the multivariate analysis.

In Brazil, the transmission of ChD by *Triatoma Infestans* is eradicated, with certification issued by the WHO in 2006. This certification is result of systematized disease control actions, structured in Brazil in 1975 [31]. Although the transmission of the disease by *Triatoma Infestans* vector is eradicated, secondary triatomines persist in the nature and can take place of the eliminated species and transmit ChD. Thus, we cannot guarantee that patients treated with BZN have not been reinfected, however, so far, no vector transmission has been identified after WHO certification. Brazil is currently in the surveillance phase, which includes the participation of the population in the notification of triatomines identified in the households. This notification is made to *Triatomine Information Centers* implanted in areas with a higher risk of reinfestation [32]

Among the strengths of the study were the large sample of ChD patients living in an endemic area, in contrast to other studies that had smaller numbers of patients from specialized clinics in large urban centers. Outcomes were measured in a reliable manner that effectively reflects the clinical conditions of all patients, as well as their parasitological status. The Genetic Matching applied in the statistical analysis is used as an approximation to traditional clinical trial for inference of causality, and its results were confirmed by traditional regression methods. Problems of regional heterogeneity of *T*. *cruzi* strains and uneven access to health system, sometimes important in other studies, are not an issue in our study, as all patients came from the same region, with similar limited access to health care services. Finally, our results are robust, as a positive and strong effect in the treated group was observed in all clinical and parasitological outcomes.

In conclusion, if used in the early phases of the disease, benznidazole treatment may result in better clinical and parasitological outcomes. Because there are millions of untreated ChD patients in the world and no new treatments are available for the foreseeable future, it is reasonable to consider treating all Chagas disease patients without advanced cardiopathy with benznidazole, especially those who are less than 50 years of age.

## Supporting information

S1 Supporting Information The questionnaires and interviewer guides. (PDF)
