# Supplementary material for: Beneficial effects of benznidazole in Chagas disease: NIH SaMi-Trop cohort study
Source: PLoS Negl Trop Dis. 2018 Nov 1;12(11):e0006814. doi: 10.1371/journal.pntd.0006814 (PMC6211620; doi:10.1371/journal.pntd.0006814)
Supplement: S1 Supporting Information — (PDF) [file pntd.0006814.s001.pdf]

SAMI TROP PROJECT

"Markers of prognostic in cronic Chagas cardiopathy"

Questionnaire I - Visit

University of São Paulo – Medical Tropical Institute
Telehealth Program: Federal University of Minas Gerais.

1.) City ____

2.) Date of interview: [__][__]/[__][__]/[__][__][__][__]
(day/month/year)

3.)Interviewer's ID number (RG):: [__][__][__][__][__][__][__][__][__]

3.a)Interviewer's Name:
[__][__][__][__][__][__][__][__][__][__][__][__][__][__][__][__][__][__]
[__][__][__][__][__][__][__][__][__][__][__][__]

Tópico I- Demographic information:

1.) Study ID:[__][__][__][__][__][__][__][__][__][__][__][__][__]

2.) Age: [__][__][__](years)

3.) Sex: [__] Male [__] Female

4.) Date of Birth: [__][__]/[__][__]/[__][__][__][__]

5.) Place of birth – City:

[__][__][__][__][__][__][__][__][__][__][__][__][__][__][__][__]

[__][__][__][__][__][__][__][__][__][__][__][__][__][__][__][__]

6.) State of Birth : [__][__]

[__][__][__][__][__][__][__][__][__][__][__][__][__][__][__][__]

[__][__][__][__][__][__][__][__][__][__][__][__][__][__][__][__]

Topic II – Subject characteristics

Now we are going to ask you some general questions:

1.) What is your color?
[__] Caucasian
[__] Black
[__] Yellow (Asian)
[__] Mulatto
[__] Indian
[__] Refused to answer

2.) What is your educational level?
[__] Adult alphabetizing
[__] Never been to school
[__] I have been to school but I did not complete Elementary school
[__] Elementary school
[__] High school
[__] College or technical school
[__] Graduate school
[__] Master degree
[__] Doctor degree
[__] Refused to answer

3.) What is your total family income? R$___________,00.
(Must be included all monthly income of all people that live in the same house of the participant)
.
[__] Refused to answer **[__] I do not know**

3.a) How many people live in your house including you?

____

FOR ALL PARTICIPANTS

Topic III- Lifestyle and Health

Now we are going to ask you some questions about your lifestyle and your current health. Please answer each question as completely as possible.

4.) Which of the following statements best describes your use of cigarettes?

[__] Never smoked (Skip to question 18)
[__] Used to smoke but don't anymore
[__] Currently smoke cigarettes
[__] Refused to answer

5.) If you currently smoke or when you used to smoke:

[__] Smoked no more than 100 cigarettes in my entire life
[__] Smoked some days, but not every day
[__] Smoked less than a pack of cigarettes every day
[__] Smoked about one to two packs of cigarettes every day
[__] Smoked at least two packs of cigarettes every day
[__] Refused to answer

6.) How many times in the last 30 days have you drunk?

[__] Every day or almost every day
[__] 3 to 5 times a week
[__] 1 to 2 times a week
[__] Less than one time a week
[__] Didn't drink in the last 30 days (skip to question 20)
[__] Refused to answer

7.) How many drinks, in average, do you use to drink?
(Obs: a drink is equal to a dose of distilled drink, a glass of beer or a glass of wine)

[__] 1 to 2
[__] 3 to 5
[__] more than 5 less than 10
[__] more than 10
[__] Don't Know
[__] Refused to answer

Physical Activity

8.) Do you participate in exercise or any physical activity where you exert yourself, such as sport? (eg.: football, tennis, running, swimming, etc..)

[__] Yes
[__] No (go to question 23)
[_ _] Refused to answer

8.a) How many days per week do you exercise or practice this activities)?

[__] 1 time per week
[__] 2 to 3 times per week
[__] 4 to 6 times per week
[__] Every day
[__] Refused to answer

8.b) How long does this activity take each time you do it?

[__] Less than 15 minutes
[__] 15 to 30 minutes
[__] 30 minutes to 1 hour
[__] 1 hour to 2 hours
[__] More than 2 hours
[__] Refused to answer

9.) Are you able to do the following each of the following physical activities:

9.1) Can you walk down a flight of 8 steps or more without stopping?

[__] Yes
[__] No
(If Yes go to 9.2, if No go to 9.4)

9.2a) Can you carry anything up a flight of 8 steps or more without stopping?

[__] Yes
[__] No

9.2b) Can you have sexual intercourse without stopping?

[__] Yes
[__] No
[__] Refused to answer

9.2c) Can you do outdoor work such as garden, rake, or weed?
[__] Yes
[__] No

9.2d) Can you slow dance?
[__] Yes
[__] No

9.2e) Can you walk at a normal pace on level ground?
[__] Yes
[__] No
(If Yes to any 9.2 question go to 9.3, if No go to question 10)

9.3a) Can you carry at least 11 kilograms (24 pounds) up a flight of 8 steps or more without stopping?
[__] Yes
[__] No

9.3b) Can you lift objects that weigh at least 36 kilograms (80 pounds)?
[__] Yes
[__] No
9.3c) Can you do outdoor work such as shovel or spade dirt?
[__] Yes
[__] No

9.3d) Can you do recreational activities such as soccer, basketball or volleyball?
[__] Yes
[__] No

9.3e) Can you walk at a face pace or jog slowly on level ground?
[__] Yes
[__] No

(Go to question 10)

9.4a) Can you stand and take a shower without stopping or sitting down?
[__] Yes

[__] No

9.4b) Can you change the sheets or remake a bed?
[__] Yes
[__] No

9.4c) Can you hang washed clothes out to dry?
[__] Yes
[__] No

9.4d) Can you clean windows, or mop floors?
[__] Yes
[__] No

(If No to any 9.4 question go to 9.5, if Yes for all the 9.4 questions go to question 10)

10.5) Can you dress yourself alone without stopping or receiving help?
[__] Yes
[__] No

11.) How do you feel physically when performing any of the following physical activities: climbing stairs, sweeping the sidewalk or home, car washing or other activities such as these?

[__] Do not feel fatigue, shortness of breath on exertion and palpitation;
[__] I feel sometimes, fatigue, shortness of breath on exertion and palpitation, but if I stop to rest or stop the activity, everything returns to normal;
[__] I feel good when at rest, but when doing any physical activity I feel shortness of breath on exertion, fatigue or palpitations;
[__] I'm uncomfortable even at rest and any physical activity makes me feel worse of shortness of breath on exertion, fatigue or palpitations.

12.) Use these codes to answer the following questions:

FOR THE INTERVIEW: SHOW TO THE PARTICIPANT THE RESPONSE CARD NUMBER 1

0 = I am not able to do
1 = I am able to do without help
2 = I am able to do with someone helping me
9 = I do not want to answer

[__] Are you able to perform heavy work in your home, such as: sweep across the yard, wash windows, wash bathrooms?

[__] Are you able to go up and down two flights of stairs without help?

[__] Are you able to walk one kilometer without getting tired? (10 blocks)

[__] Would you be able to do all the housework alone? Do all washing and cleaning?

[__] Could you cook?

[__] Would you be able to shop at the market and supermarket?

Topic IV - ANAMNESIS (Basic Medical History)

13.) Have you been told by a doctor or health professional that you have high cholesterol or cholesterol problems?
[__] Yes
[__] No (Skip to question 27)
[__] Don't Know
[__] Refused to answer

14.) If yes, have you been under medical treatment (taking medicine) for high cholesterol?
[__] Yes
[__] No
[__] Don't Know
[__] Refused to answer

15.) Have you been told by a doctor or health professional that you have tegumentar or visceral leishmaniasis, generally known as KALAZAR?
[__] Yes
[__] No (go to question 16)
[__] Don't Know
[__] Refused to answer

15.a) If YES, how long did you have this disease?

__________________ Months/Years (mark the correct option according the time the patient informed)

16.) Have you been told by a doctor or health professional that you have Chagas disease?
[__] Yes
[__] No
[__] Don't know
[__] Refused to answer

16.a) If YES, how long have ago were you diagnosed with Chagas disease?
[__] Less than 1 year
[__] 1 to 5 years
[__] 5 to 10 years
[__] More than 10 years ago
[__] Don't know
[__] Refused to answer

17.) Are you under medical care for Chagas disease at this time?
[__] Yes
[__] No
[__] Don't know
[__] Refused to answer

17.a) If YES, can you tell us where?

[__] In this Health Unit (the same Unit Health where the interview is been conducted)
[__] Another place
[__] Refused to answer

17.b) Name of place ____________________________________

TOPIC V. TREATMENT

18.) Have you ever take the medicine called ROCHAGAN OR BENZONIDAZOL?

[__] Yes
[__] No (go to question 25)
[__] Don't know
[__] Refused to answer

If YES, please answer the following questions.

19.) How long ago did you take this medicine for the FIRST TIME?

[__] days (go to question 20)
[__] months (go to question 20)
[__] years (go to question 20)
[__] I do not know (go to question 20)
[__] Refused to answer (go to question 20)
[__] I am taking this medicine actually (go to question 19a.)

19.a) I have been taking this medicine since ___/___/____.

20.) What was the duration of your first treatment?
[__] days
[__] months
[__] years
[__] I do not know
[__] Refused to answer (go to question 21)

21.) What was the dose of the first treatment?

FOR THE INTERVIEWER: We need to know this information as detailed as possible. If the patient knows the number of tablets and the dose in milligrams, fill up both information.

[__] tablets per day
[__] mg per day
[__] I do not know
[__] Refused to answer

22.) How long after the diagnostic did you take this medicine?

[__] Immediately or less than one month
[__] months
[__] years
[__] I do not know
[__] Refused to answer

23.) Did you take this medicine more than one time?

FOR THE INTERVIWER: THIS QUESTION REFERS TO ANOTHER TREATMENT BESIDES THE FIRST ONE.

[__] Yes
[__] No (go to question 24b)
[__] Don't know
[__] Refused to answer

24.a) If YES, how many times did you take this medicine?

______ times.

24.b) When was the last time that you took this medicine?
[__] months ago
[__] years ago
[__] I do not know
[__] refused to answer

25.) Have you ever taken the medicine called AMIODARONE or ATLANSIL?
[__] Yes
[__] No (go to question 28)
[__] Don't know
[__] Refused to answer (go to question 28)

If YES, please answer the following questions.

26.) How long did you take this medicine?
[__] days
[__] months
[__] years
[__] I do not know (go to question 27)

[__] Refused to answer (go to question 27)

27.) What was the dose of this medicine that you took?

FOR THE INTERVIEWER: We need to know this information as detailed as possible. If the patient knows the number of tablets and the dose in milligrams, fill both information.

[__] tablets per day
[__] mg per day
[__] I do not know
[__] Refused to answer

28.) Have you been told by a doctor or health professional that you have some of these health problems listed below?

CODES	1=Yes<br>2=No<br>3=do not know<br>4= Refused to answer
Diabetes	
Kidney problems	
Problems with blood clotting	
Myocardial infarction	
High blood pressure	
Thyroid disease	

29.) Identify from the below signs or symptoms the one or ones you have or have had:

	YES	NO
29.a) Do you feel pain when you swallow food?		
29.b) Do you have trouble swallowing or have the sensation of "food grasping" in your esophagus?		
29.c) Has a doctor, based on a chest X-Ray, told you that you have a big esophagus?		
29.d) Is it usual for you to have no bowel movement for 3 or more days? How many times has this happened to you in the last month? ________		
29.e) Has a doctor, based on a abdominal X-Ray, told you that you have a big colon?		

29.f) Do you feel unable to climb 2 flights of stairs without resting?		
29.g) Do you have heart palpitations?		
29.h) Do you have difficulty breathing when lying down?		
29.i) Have you ever felt prolonged faintness or dizzy?		
29.j) Have you experienced swelling or puffiness in your feet in the morning?		
29.k) Does your heartbeat sometimes race when resting?		
29.l) Have you noticed any visible neck veins in the mirror when standing up or sitting?		
29.m) Has your doctor told you that you have heart abnormalities or rhythm problems on electrocardiogram?		
29.n). Have you been told that your heartbeat is not regular?		
28.o) Do you have permanent pace maker insertion?		
29.p) Have you ever noticed your heartbeat racing or beating abnormally?		
29.q) Have you ever awaked during the night short breathed or not being able to breath?		
29.r) Do you feel short breath when you have to use physical strength, for example: climbing stairs or hills?		

30.) Do you take any of these drugs?

**Medicine**	**YES**	**NO**	**Time of use (years)**
Digoxina			
Furosemida			
Hidroclorotiazida			
Captopril			
Enalapril			
Losartan			
Espironolactona			
Carvedilol			

Hydralazine			
Mononitrato de isosorbide amlodipina			
Warfarin			
AAS			
Atenolol			
Propanolol			

31.) Have you ever had or have fainting or loss of consciousness?
[__] Yes
[__] No (go to question 32)
[__] I do not know
[__] Refused to answer

31.a) If YES, how many times it happened in the last two years?
[__][__][__] times
[__] I do not remember
[__] Refused to answer

31.b) Do you remember how long in average did each episode take?
[________] minutes
[__] I do not remember
[__] Refused to answer

32.) Do you monitor your health in another service for other medical illness?
[__] Yes, just once
[__] Yes, more than one time
[__] No
[__] Refused to answer

33.) How do you rate your health condition today?
[__] Very good
[__] Good
[__] Average
[__] Bad
[__] Very bad
[__] I do not know
[__] Refused to answer

34.) Besides you, someone else in your family has or had Chagas disease?

[__] Yes
[__] No
[__] I do not know
[__] Refused to answer

34.a) If yes, what degree of kinship (father, mother, brother, sister, uncle, aunt, etc.): ________________

Thank you for participating in this International Research Project. Be sure that you are contributing to improve knowledge about Chagas Disease and quality of care provided to patients.

35.) Quality of life. WHOQOL-Bref.

FOR THE INTERVIEWER: This module is based on subjective perception of patient. Only he/she can answer these question
